# Quantification of DNA double‐strand breaks using Geant4‐DNA

**DOI:** 10.1002/mp.13290

**Published:** 2018-12-07

**Authors:** Konstantinos P. Chatzipapas, Panagiotis Papadimitroulas, Mohammad Obeidat, Kristen A. McConnell, Neil Kirby, George Loudos, Niko Papanikolaou, George C. Kagadis

**Affiliations:** ^1^ Department of Medical Physics University of Patras Rion GR 26504 Greece; ^2^ R&D Department BioEmission Technology Solutions Athens GR 11472 Greece; ^3^ University of Texas Health Science Center San Antonio TX 78229 USA; ^4^ University of West Attica Department of Biomedical Engineering Egaleo GR 12243 Greece

**Keywords:** DNA dosimetry, DNA double‐strand breaks, Geant4‐DNA, Monte Carlo simulations, radiobiology

## Abstract

**Purpose:**

This study aims to standardize the simulation procedure in measuring DNA double‐strand breaks (DSBs), by using advanced Monte Carlo toolkits, and newly introduced experimental methods for DNA DSB measurement.

**Methods:**

For the experimental quantification of DNA DSB, an innovative DNA dosimeter was used to produce experimental data. GATE in combination with Geant4‐DNA toolkit were exploited to simulate the experimental environment. The PDB4DNA example of Geant4‐DNA was upgraded and investigated. Parameters of the simulation such energy threshold (ET) for a strand break and base pair threshold (BPT) for a DSB were evaluated, depending on the dose.

**Results:**

Simulations resulted to minimum differentiation in comparison to experimental data for ET = 19 ± 1 eV and BPT = 10 bp, and high differentiation for ET<17.5 eV or ET>22.5 eV and BPT = 10 bp. There was also small differentiation for ET = 17.5 eV and BPT = 6 bp. Uncertainty has been kept lower than 3%.

**Conclusions:**

This study includes first results on the quantification of DNA double‐strand breaks. The energy spectrum of a LINAC was simulated and used for the first time to irradiate DNA molecules. Simulation outcome was validated on experimental data that were produced by a prototype DNA dosimeter.

## 1 Introduction

Ionizing radiation can have both positive and negative influence on peoples’ health. It leads to genetic modifications that unless repaired, can ultimately lead to cell death. Every day, because of the oxidative stress, every human cell experience more than 50,000 lesions that could lead to DNA mutations through reactive oxygen species (ROS),^1^ produced by the aerobic metabolism. A dose of 2 Gy results to only ~3000 DNA lesions.^2^ DNA‐double‐strand break (DSB) is reported in literature as a dominant factor for maleficent lesions produced by ionizing radiation, because even a single unrepaired one can cause cell death.^3^, ^4^, ^5^, ^6^ DSB is called a break in the phosphodiester backbone of both DNA strands, separated by 10 base pairs (bp).^7^, ^8^, ^9^, ^10^, ^11^, ^12^, ^13^ The number of DSBs increases linearly with dose.^14^

Radioprotection is a constant concern to our society, for people either working on health care, in a power plant, near a particle accelerator, or even in the daily life by being exposed to environmental radiation. But, there is not a clear way to estimate health hazards from those exposures because of the lack on data at such low doses. Biological effects are estimated with mathematical models, adjusted by extrapolation of data collected from the atomic bombs of Hiroshima and Nagasaki. Consequently, there is no safe way to estimate the true risk of the human's health to low‐dose exposure to ionizing radiation.^15^

Along the last decades several Monte Carlo (MC) codes have been developed for the investigation of ionizing interactions with matter.^16^ Lately, many MC codes such as Geant4,^17^ PARTRAC,^18^ KURBUC‐liq^19^ include the physicochemical, and chemical stage of the interactions, as well as the modeling of DNA molecule for radiobiology applications.^20^ In addition, TOPAS‐nBio^21^ has the same capabilities by being a Geant4‐DNA‐based user code. In the early ‘00s Dr P. Nieminen, proposed the estimation of biological effects produced by cosmic radiation.^22^ In 2010, the geant4‐dna project (http://geant4-dna.org) was established to develop such a platform for the simulation of molecular and cellular radio‐biological interactions including physicochemical properties.^15^ Nowadays, Geant4‐DNA is a standard tool for studying low scale simulations as it includes physics and chemistry modeling for low‐energy processes applicable in the DNA nano‐level, as well as in nano‐dosimetry.^23^

The Geant4‐DNA code is prototype but there is a big effort to be standardized and validated.^24^, ^25^ Furthermore, new experimental and validation studies need be performed to assess its accuracy.^17^

Several studies experimentally assessed DNA‐DSBs for dosimetry measurements.^26^, ^27^ In this study, the newly collected experimental data from Obeidat et al.*,* 28 on the quantification of DNA‐DSBs are used to validate Geant4‐DNA physical models on nano‐dosimetry. The default algorithm of Geant4‐DNA example (PDB4DNA) for DSB quantification^25^ was further studied and edited using advanced C++ tools. The outcome was compared with experimental data. This is a substantive extension of the authors’ work on a preliminary abstract that has been presented previously.^29^

There have been several studies that investigate DNA‐DSBs. H. Nikjoo et al. used 17.5 eV at their Monte Carlo study to model the damage by direct deposition and 10 bp to produce a DSB.^30^ M. Huels et al. proposed 10 eV as the energy threshold for DSB within 10 bp.^31^ Y. Huang et al. also used 10 bp as the distance that separates two single‐strand breaks and define a DSB, the same parameter was used by V. Semenenko et al. and R. Stewart et al.^32^, ^33^, ^34^ Furthermore, S. Meylan et al. also used 17.5 eV and 10 bp.^35^

## 2 Methods and materials

The experiments to quantify DNA‐DSBs were performed by coauthors at the UT Health San Antonio using a prototype dosimeter which is described elsewhere.^28^, ^36^ The DNA dosimeter contains linear double‐stranded DNA (dsDNA) segments suspended in phosphate‐buffered saline (PBS). These DNA strands are tagged with fluorescein amidite (6FAM) and biotin on opposing ends. The biotin end binds the strands to magnetic streptavidin beads. When a DSB happens, the strand is cut in two pieces: the bead/biotin end and the 6FAM end (fluorescing broken ends). To separate the uncut pieces from the damaged ones, a magnet is used. The two different samples are read in a fluorescence reader. The probability of double‐strand break, P(DSB), is defined here as the fraction of DNA strands that receive one or more DSB. The relative fluorescence of the broken over the unbroken dsDNA is utilized to determine this probability.

Three different experiments were performed to irradiate the DNA dosimeter samples with 6 MV photons from a Clinac 600 C/D linear accelerator (Varian, Palo Alto, CA, USA) to 25, 50, 100, 150, and 200 Gray (Gy). Each dosimeter consisted of 50 μl of solution held in a 1.5 ml microcentrifuge tube. These tubes were placed inside a water tank (MP3, PTW, Freiburg, Germany) at a 5 cm depth and at 65 cm distance from the source. Before irradiating the dosimeters, a calibrated 0.3 cm^3^ Semiflex 31013 ionization chamber (PTW, Freiburg, Germany) was utilized along with the AAPM Task Group 51 calibration protocol^37^ to determine absorbed dose during the experiment.

The experimental setup was simulated with the Geant4‐DNA code. The DNA dosimeter contains linear double‐stranded DNA (dsDNA) suspended in phosphate buffered saline (PBS). The concentration specification for the PBS used is 1X, the pH is 7.4, and the osmolarity is 280–315 mOsm/kg. PBS is a solution that contains scavenger that is very keen on interacting with free radicals.^38^, ^39^ This way, DNA molecules are protected to some extent, by the OH^−^ produced through water radiolysis. This fact means that an approach using only physical interaction can produce results close to the experimental ones. Τo quantify the contribution of the indirect damage further investigations on the DSB yield change with PBS concentration would be required, which was outside the aim of the present study. Accurate modeling of the energy spectrum that irradiates the DNA molecule plays a crucial role to reproduce the experimental measurements and standardize the simulated results.

To irradiate the DNA molecule with a realistic electron spectrum which derived from the LINAC X‐ray spectrum, we used GATE MC toolkit and the phase‐space (PhSp) concept. GATE was selected as it was extensively validated by coauthors.^40^, ^41^, ^42^ More precisely, a validated 6 MV photon energy spectrum^43^ was modeled in GATE, irradiating a PBS volume, equal to 30 × 30 × 30 cm^3^ for the purpose of producing electronic equilibrium. The Penelope electromagnetic physical processes were enabled to model the physical interactions within the PBS. A PhSp of rectangular volume (0.1 × 0.1 × 0.1 cm^3^) was attached to the PBS volume. 5·10^8^ primaries (photons) were used for the generation of 4·10^6^ electrons. The PhSp was investigated using ROOT and a C script to extract the information related to electrons. Using this PhSp, an electron spectrum was produced to be used as the radiation source for the simulation of DNA irradiation, reproducing the electronic equilibrium. The DNA molecule was irradiated isotropically, to produce the electronic equilibrium and the same Linear Energy Transfer (LET) as in the real experiment. GATE v7.2^44^ alongside with Geant4‐10.2,^45^ and ROOT v5.34^46^ were used.

**Figure 1:**
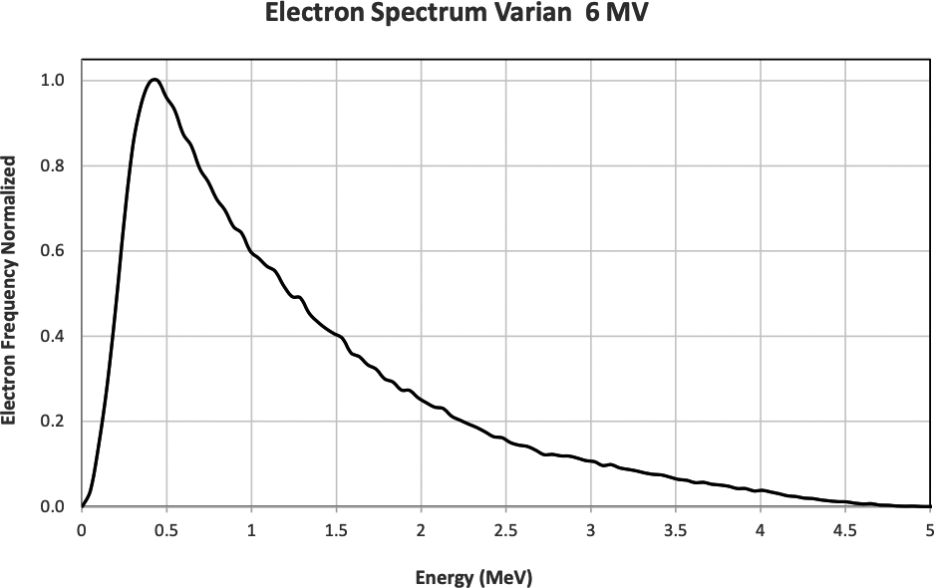
Electron spectrum calculated in a phase‐space through the interaction of 6 MV photons with PBS (GATE simulation).

Geant4 release 10.3 was used to model the DNA molecules and the DNA physics interactions with respect to the experimental properties.^47^, ^48^ More specifically, an updated version of the PDB4DNA example was investigated, the PDB4DNA_v0, where the quantification of the DSBs is reviewed. A new class was designed to insert the general particle source concept and to integrate the electron spectrum (shown in Fig. 1). In the appendix, the upgraded code for measuring DNA SSB and DSB is presented. G4EmDNAPhysics model was used for the implementation of all the simulations. The program's algorithm was also upgraded, to create multiple outputs when running on a cluster.

In the present study, 52% percent of the electrons’ energy is <1 MeV, as seen on Fig. 1. Electrons with energy >1 MeV have an electron range >0.4 cm, as documented in literature.^16^, ^49^, ^50^ The 1ZBB DNA molecule dimensions are 12 × 15 × 25 nm. Hence, for the dimensions speaking in the current study, we could consider the contribution of the electrons with energies >1 MeV to the energy deposition negligible. This is a limitation of the study and should be an objective of future research.

An in‐house batch file was developed to give the user the opportunity to insert several parameters that affect the simulation. The user can take advantage of it, to easily modify the irradiation spectrum, the seed number, the energy threshold (ET) for a DNA strand break on the phosphodiester bond, as well as the base pair threshold (BPT) that defines the distance between two different strand breaks on opposite strands that produce a DSB, without the need of prior C++ programming knowledge.

**Figure 2:**
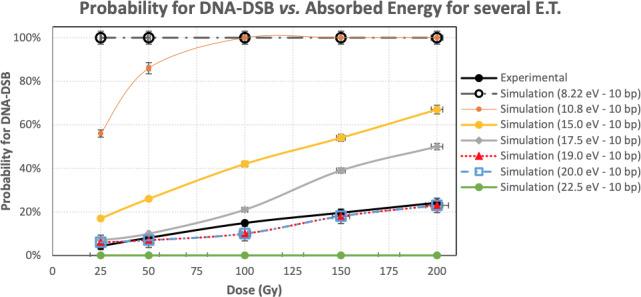
Probability for DNA‐DSB for several Energy Threshold, depending on the Dose in the DNA molecule. [Color figure can be viewed at wileyonlinelibrary.com]

Several values were investigated for the energy threshold ranging from 10 to 22.5 eV and the distance threshold ranging from 6 to 14 bp.^30^, ^31^, ^32^, ^33^, ^34^, ^35^ Several studies are using different values for the assessment of DSBs in molecular level; however, there is not a standardized value till now. The impact of these parameters is meticulously studied about their relation, and each one separately.

A batch file was also used, in connection with a Python script, to create a population of different simulations, to be uploaded on a cluster. For the simulations acquired in this study, the national High‐Performance Computing (HPC) Infrastructure “ARIS” was used.^51^

The Research Collaboratory for Structural Bioinformatics Protein Data Bank was exploited^52^ to model the DNA molecule, which was a tetranucleosome (“1ZBB”). Classes already integrated in the PDB4DNA example were used for its integration in the program.^25^

For every separate experiment with different absorbed energy 1000 different simulations were performed, while changing the initial seed number (control theory), for statistical reasons (*σ *= 0.009). To achieve the exact experimental doses, we used 3·10^5^ primaries for the 25 Gy simulation up to 2.4·10^6^ primaries for the 200 Gy case (absolute dosimetry).

The ROOT data analysis framework was used to estimate the deposited energy (in MeV) on the volume of the DNA molecule, as well as any single‐ and double‐strand break. Statistical analysis was performed, to extract the probability for a DSB as a function of the deposited energy and consequently absorbed dose using the Protein Data Bank information.

## 3 Results

The data obtained with the DNA dosimeter are presented in Table 1, alongside with their standard error of the mean (SEM) for each measurement. SEM is given by the following equation:

(1) $$SEM=\frac{\mathrm{SD}}{\sqrt{N}}$$

where, SD is the Standard Deviation of the population, and N is the size of the sample.

**Table 1:** Experimental data on DNA double‐strand break probability

Dose (Gy)	Experimental probability for DSB	SEM
25	4.3%	0.2%
50	8.1%	0.3%
100	14.9%	0.6%
150	19.6%	0.5%
200	24.2%	1.1%

The experimental probability of DNA‐DSBs, as it was measured using the DNA dosimeter, is depicted in Fig. 2 and is following the linear function in Eq. (2):

(2) $$P\left(\mathrm{DSB}\right)=-6\cdot 10^{-6}\cdot D^{2}+1.7\cdot 10^{-3}\cdot D+3.9\cdot 10^{-3}$$

**Figure 3:**
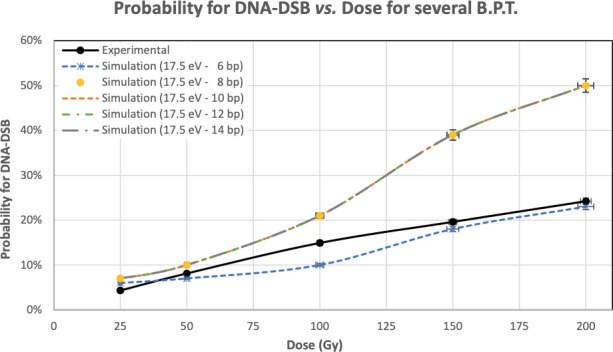
Probability for DNA‐DSB for several Base Pair Threshold, depending on the Dose in the DNA molecule. [Color figure can be viewed at wileyonlinelibrary.com]

As, the purpose of this study was to investigate and standardize the Geant4‐DNA simulation procedure for quantifying DNA‐DSBs, we studied several cases with different combinations of energy and distance thresholds. In every simulation, we used the same electron spectrum but different parameters for ET and BPT.

The probability for a DNA‐DSB is depicted in Fig. 2, in relation to several ET for a strand break. The distance between two breaks that produce a DSB was set to 10 bp, which is the most frequently used value in the literature. The simulation output is very close to the experimental data when the ET is set at the values of 18–20 eV. The default value of 8.22 eV, proposed in the PDB4DNA example provides 100% probability for DSB for any dose. 10.79 eV, proposed in literature^53^ also gives DNA‐DSB probability higher than the experimental one. 19 ± 1 eV shows the best agreement to the experimental measurements, with differences ranging from 1% to 5%.

To further investigate the simulated results using PDB4DNA, 17.5 eV value was set to all simulations, while the bp distance was modified Fig. 3.

**Figure 4:**
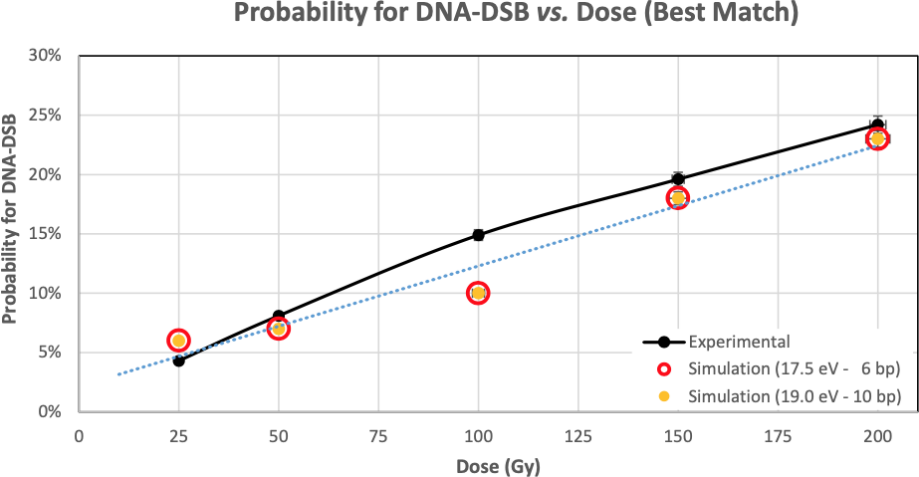
Probability for DNA‐DSB depending on the Dose in the DNA molecule. [Color figure can be viewed at wileyonlinelibrary.com]

The investigation has been extended to study the influence of the BPT. Keeping ET for a strand break at 17.5 eV (value proposed in Ref. 35) and changing the distance for having DSB. This study evaluates the values of BPT between 6 and 14 bp to compare the simulated DSB percentage with the experimental one.

Based on the experimental data, a couple of the ET and BPT values on the simulated results provided accurate DNA‐DSBs, with differences lower than 5% for 19 ± 1 eV and 10 bp, and 5% for 17.5 eV an 6 bp, as presented in Fig. 4.

## 4 Discussion

The DNA dosimeter measures any damage that leads to the separation of 6FAM edge of the DNA with the streptavidin edge. This means that it may record as a DSB either a simple break at the connection of 6FAM with the DNA, or a clustered damage to the DNA molecule. At this point, it cannot separate the different mechanisms that lead to the separation of the edges. Moreover, multiple breaks may exist on a DNA molecule but it will only count one DSB instead of a complex DSB. It measures if the DNA molecule is broken or not, independently of the number of cuts. An analytical description of the dosimeter has recently been reported in the study by Obeidat et al.^28^ The same approach is also followed in the simulation. It only records whether a molecule is destroyed or not.

Geant4‐DNA simulations tend to be a standard tool for studying the molecular biological effects and DNA damage. As already reported in literature, there are several limitations in such a low scale dosimetry that need be addressed. The major issue for optimizing the simulated models is the lack of experimental data, when trying to evaluate these procedures. In the proposed study, novel experimental dosimetry data were used for the DNA‐DSB quantification.

For the simulation study, we used the PDB4DNA example of Geant4‐DNA, incorporating upgrades on the loops on the DSB quantification. The edited PDB4DNA_v0 code includes: (a) corrections on the loops for counting SSBs and DSBs, (b) integration of General Particle Source concept, (c) integration of cluster option for running and analysis.

The proposed study shows that Geant4‐DNA simulations provide an accurate outcome in modeling the DNA‐DSB probability, in accordance with experimental data (Fig. 4). Based on the literature, this study proposes that it is safe to use 18–20 eV as the energy threshold for a DNA strand break and 10 bp as the distance between two different strand breaks on opposite strands that define a DSB, when using G4EmDNAPhysics. This setup gives a differentiation up to 5%, while for ET 17.5 eV the differentiation is between 10% and 25%, and for ET <15 eV the differentiation is higher than 20%. For ET >22.5 eV the DSB probability is 0% for any dose. Moreover, there is a connection of the experimental data and the simulation, when using 17.5 eV and 6 bp, but no such threshold for base pairs is recommended in the literature.

Furthermore, it must be stated that recently published articles have reported more accurate physics models for low‐energy electron tracking and DNA damage than the G4EmDNAPhysics (the G4EmDNAPhysics_option4).^48^, ^54^, ^55^ We are planning to investigate the impact of these models on the DNA‐DSB quantification. Further investigation is needed to study the effect of chemical interactions, when the two different sets of parameters are used.

This study extends the knowledge on DNA dosimetry and the validity of the corresponding simulations. Electron energy spectrum is used for the first time instead of monoenergetic particles. That information can be used and extend to provide the biological result that several types of irradiation produce to the human tissues. An initial correlation between deposited energy on DNA and the creation of DSBs in DNA molecule was achieved. Moreover, the parameters of (a) the energy threshold for a DNA strand break and (b) base pairs for DSB has been analyzed.

## 5 Conclusion

In conclusion, more research is required for the evaluation of the DNA dosimeter as well as on the simulation models. A more accurate model of the exact experiment is needed, irradiating multiple DNA molecules, instead of a single one. The integration of chemical interaction is also of high importance. By this integration, it will be possible to quantify the proportion of DSBs derived by physical interactions compared to the ones derived by water radiolysis (chemical species – free radicals), and could be a research quest of a future study. Finally, the simulation model as much as the DNA dosimeter need to be fully tested in low‐dose cases (<25 Gy), to be able to evaluate clinical cases of irradiation.

## Conflicts of interest

The authors have no conflicts to disclose.
